# Global priorities for national carnivore conservation under land use change

**DOI:** 10.1038/srep23814

**Published:** 2016-04-01

**Authors:** Enrico Di Minin, Rob Slotow, Luke T. B. Hunter, Federico Montesino Pouzols, Tuuli Toivonen, Peter H. Verburg, Nigel Leader-Williams, Lisanne Petracca, Atte Moilanen

**Affiliations:** 1 Department of Biosciences, University of Helsinki, FI-00014, Helsinki, Finland; 2 School of Life Sciences, University of KwaZulu-Natal, Durban 4041, South Africa; 3 Department of Genetics, Evolution and Environment, University College, London, United Kingdom; 4 Panthera, New York, New York, United States of America; 5 Rutherford Appleton Laboratory, Science & Technology Facilities Council, Harwell Oxford Campus, Didcot OX11 0QX, UK; 6 Department of Geosciences and Geography, University of Helsinki, FI-00014, Helsinki, Finland; 7 Faculty Earth and Life Sciences, VU University Amsterdam, De Boelelaan 1087, Amsterdam, 1081 HV, The Netherlands; 8 Department of Geosciences and Geography, University of Cambridge, Downing Place, Cambridge, CB2 3EN, UK

## Abstract

Mammalian carnivores have suffered the biggest range contraction among all biodiversity and are particularly vulnerable to habitat loss and fragmentation. Therefore, we identified priority areas for the conservation of mammalian carnivores, while accounting for species-specific requirements for connectivity and expected agricultural and urban expansion. While prioritizing for carnivores only, we were also able to test their effectiveness as surrogates for 23,110 species of amphibians, birds, mammals and reptiles and 867 terrestrial ecoregions. We then assessed the risks to carnivore conservation within each country that makes a contribution to global carnivore conservation. We found that land use change will potentially lead to important range losses, particularly amongst already threatened carnivore species. In addition, the 17% of land targeted for protection under the Aichi Target 11 was found to be inadequate to conserve carnivores under expected land use change. Our results also highlight that land use change will decrease the effectiveness of carnivores to protect other threatened species, especially threatened amphibians. In addition, the risk of human-carnivore conflict is potentially high in countries where we identified spatial priorities for their conservation. As meeting the global biodiversity target will be inadequate for carnivore protection, innovative interventions are needed to conserve carnivores outside protected areas to compliment any proposed expansion of the protected area network.

Current rates of anthropogenic-related extinctions are unprecedented and difficult to halt^1^. Species extinction rates are now 1,000 times higher than the normal rate before humans became a primary contributor to extinctions^2^. Across vertebrates, 16 to 33% of species are considered to be globally threatened^3^. This biodiversity ‘crisis’ is driven by factors, such as agricultural expansion, logging, overkill by humans, climate change, and invasive alien species^4^. Agricultural expansion is the most frequent threat to terrestrial and inland water species, threatening 69% of species^5^. Studies suggest that extinction rates could worsen in absence of conservation action for species currently threatened with extinction^3^,^6^. Protected areas remain the cornerstone for the conservation of biodiversity^7^,^8^. However, the current protected area network fails to represent all biodiversity that conservationists believe needs protection^9^,^10^,^11^.

Mammals are severely affected by the extinction crisis, with around a quarter of extant species considered to be threatened with extinction^3^,^5^. Worryingly, the extinction risk of many mammal species has accelerated over the last 40 years^12^. Mammalian carnivores, especially the largest terrestrial species, have experienced substantial population declines, and are among the most persecuted species that have suffered the biggest range contraction among all biodiversity^13^. Carnivore declines are driven mainly by the loss or degradation of habitat and prey base, persecution by humans, as well as over-utilization (e.g. for traditional medicine or sport hunting)^5^,^14^. Biological traits, such as large body sizes, large area requirements, low densities, and slow population growth rates, make mammalian carnivores particularly vulnerable to habitat loss and fragmentation^15^. As a result, there is need to develop studies that identify priorities for mammalian carnivore conservation while accounting for landscape connectivity in order to enhance dispersal^16^.

The Convention on Biological Diversity has adopted 20 headline targets as part of its new Strategic Plan to address the biodiversity ‘crisis’^17^. For example, Aichi Target 11 recommends that at least 17% of all terrestrial land and inland water should be conserved through ecologically representative and well-connected systems of protected areas by 2020. Global spatial conservation prioritization assessments to inform such targets have shown how limited resources could be allocated to maximize species representation and reverse biodiversity decline^9^,^10^,^11^. Current gaps in research exist on how to enhance connectivity between protected areas in order to facilitate species movements and enhance gene flow between populations^8^,^18^. No comprehensive study exists that has investigated how well carnivore species will be represented within the 17% land target for global protected area expansion, while accounting for connectivity and expected agricultural and urban expansion. In addition, no previous global analysis has tested how protecting carnivores could potentially extend protection to other biodiversity via the umbrella effect. Finally, it is crucial to assess which social, economic and political factors could potentially constrain carnivore conservation in priority areas for conservation action. These are the challenges we address here.

In this paper, we used spatial conservation prioritization tools^19^,^20^ in combination with global species range maps of carnivores^5^ and global land use change scenarios^21^ to identify global spatial priorities for carnivore conservation under future land use change scenarios. Our analysis considered a total of 317 placental (e.g. lion, *Panthera leo*) and marsupial (e.g. numbat, *Myrmecobius fasciatus*) carnivore species (hereafter carnivores), by using updated species range maps for the Felidae and species range maps provided by the International Union for the Conservation of Nature (IUCN)^5^. In the analysis, we (i) evaluated the representation of all carnivore species within the global protected area network; (ii) assessed what the carnivore species representation would be if the 17% land target for global protected area expansion was allocated to carnivore conservation, both currently and under future land use change scenarios; (iii) tested the effectiveness of carnivores as umbrella species for 23,110 species of amphibians, birds, mammals and reptiles and 867 terrestrial ecoregions, both currently and under future land use change scenarios; and (iv) assessed which social, economic and political factors could potentially constrain carnivore conservation in countries that were identified as priorities for carnivore conservation.

## Results

**Figure 1:**
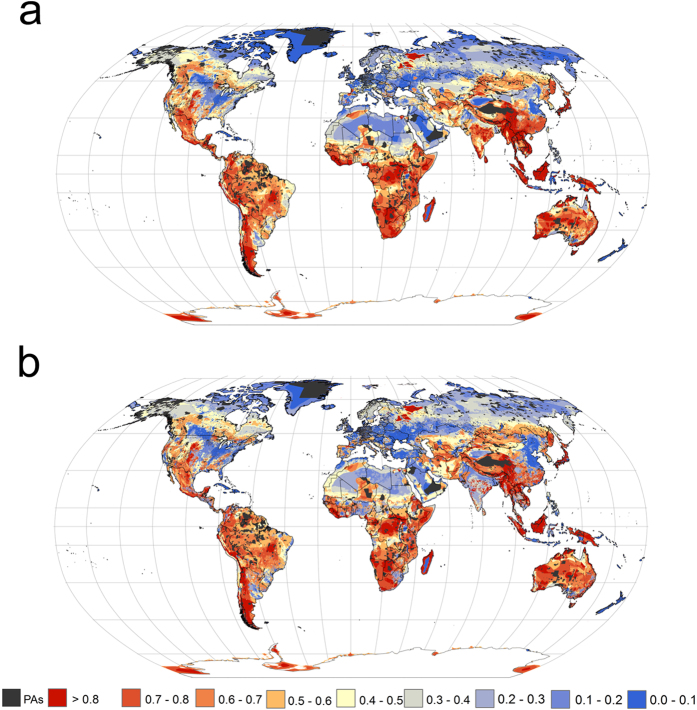
Global priority maps for the expansion of the protected area network for mammalian carnivores, by accounting for (**a**) present and (**b**) future (2040) land use change. Areas in dark red are priorities for protected area expansion. PAs = protected areas. Figure created in ArcGIS 10.2.1 software (URL http://desktop.arcgis.com/en/).

Globally, the highest present priorities for carnivore conservation outside of the existing protected area network are in South America, sub-Saharan Africa, South East Asia, Australia, and North West Russia (Fig. 1a). The spatial priorities for carnivore conservation will shift under expected land use change (Fig. 1b). At the 17% land target, the spatial overlap between the present and the future priorities for conservation action was 72.9%.

Besides shifting spatial conservation priorities, expected land use change will lead to a potential range loss of ~18% across all carnivore species (Table S1, Supplementary Results). Critically Endangered, Vulnerable, and Data Deficient species, as well as large-bodied and placental carnivores, will lose more range (Table S1, Supplementary Results). Among the most affected species, small-bodied carnivores that are endemic or near-endemic to India will suffer the biggest range losses (Table 1). Overall, the species that are mostly affected by habitat loss have already shown declining population trends, and are also affected by other threats, particularly killing by humans^5^ (Table 1).

**Figure 2:**
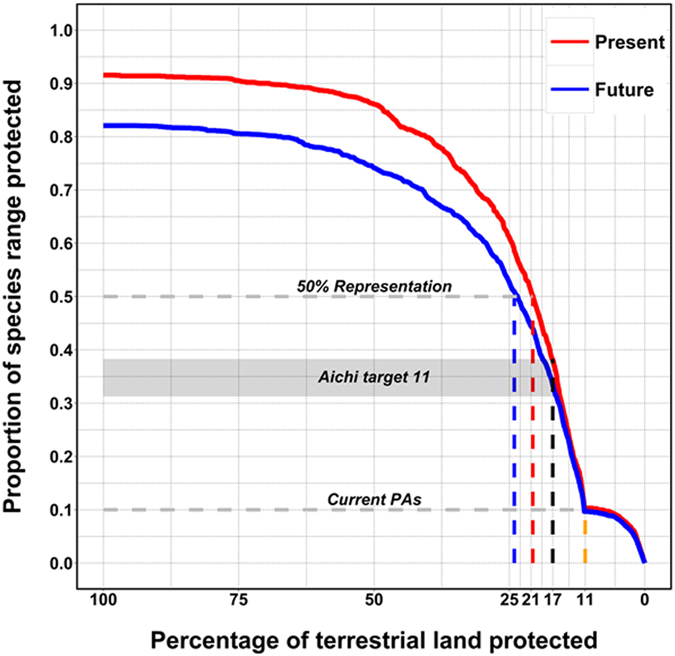
Performance curves quantifying the median proportion of the original occurrences of all carnivore species, represented at each fraction of the terrestrial land protected for carnivores. The dashed vertical line in yellow represents the percentage currently protected (~11% of terrestrial land). The vertical dashed line in black represents the 17% target for the optimized expansion of the protected area network. The dashed vertical lines in red and blue represent the terrestrial land targets required to meet a 50% representation across all carnivore species under present, and future (2040), land use allocation (21 and 24% of terrestrial land, respectively). The grey dashed lines and rectangle show the corresponding representation levels for already existing protected areas and the Aichi target 11 for 17% terrestrial land protection.

Expected land use change will also decrease carnivore representation at the 17% land target. Currently, the median proportion of carnivore ranges represented within the global protected area network is ~10% (Fig. 2). At the 17% land target, the median representation for all carnivore species would increase, but would still be lower than 40% and would decrease from present to future conditions (Fig. 2). Importantly, achieving a more adequate representation of at least 50% of the species ranges would require more land than the actual 17% proposed land target under both scenarios (Fig. 2). Threatened (i.e. the IUCN Categories of Vulnerable, Endangered, and Critically Endangered) and Data Deficient species showed the biggest drop in representation from present to future conditions (Figure S1, Supplementary Results). Larger and more predatory species among the Ursidae, Felidae, Canidae, and Hyaenidae, which have the most extensive habitat requirements, had the lowest representation (Figure S2, Supplementary Results). The median representation of the 31 largest carnivore species^13^, which have the largest extent of occurrence, would drop to <25% under the future land use change scenarios (Figure S3, Supplementary Results). Placental carnivores had overall lower representation levels than comparatively narrow-range marsupial carnivores (Figure S4, Supplementary Results).

Under present conditions (Fig. 1a), at the 17% target for terrestrial land protection, carnivores performed best as surrogates for amphibians with a median representation of ~50% of amphibian ranges (Figure S5a). The representation for terrestrial ecoregions was the lowest among the targeted groups (Figure S5a). Under expected land use change (Fig. 1b), at the 17% target for terrestrial land protection, carnivore representation for amphibians dropped by 10%, while the representation for birds, mammals and reptiles decreased less (Figure S5a). Under present conditions (Fig. 1a), at the 17% target for terrestrial land protection, carnivores performed best as surrogates for threatened (Vulnerable, Endangered and Critically Endangered) birds, amphibians, mammals and reptiles than they did for common birds, amphibians, mammals and reptiles (Figure S5b). Under expected land use change (Fig. 1b), at the 17% target for terrestrial land protection, carnivore surrogacy for threatened bird, amphibian, mammal and reptile species also decreased, with the largest drop in representation occurring for endangered (10% decrease) and critically endangered (~14% decrease) bird, amphibian, mammal and reptile species (Figure S5b).

According to the generalized linear models (Table S2 and Figure S6, Supplementary Results), the best overall predictor explaining the contribution of each country to carnivore conservation under both present and future conditions was the GINI index, which is intended to represent the income distribution of a nation’s residents and is the most commonly used measure of inequality (Table S3, Supplementary Methods). The coefficient for the GINI index had a positive sign (Figure S6, Supplementary Results), indicating that the priority areas for carnivore conservation are found in countries where the inequality levels are the highest (Table S3, Supplementary Methods). The second most important predictor was the human development index (Table S2 and Figure S6, Supplementary Results), which is a summary measure of average achievement in key dimensions of human development (a long and healthy life, being knowledgeable and have a decent standard of living) (Table S3, Supplementary Methods). The coefficient for human development index had a negative sign (Figure S6, Supplementary Results), indicating that the priority areas for carnivore conservation are found in countries where the development levels are the lowest (Table S3, Supplementary Methods). The explanatory power of these models (the percentage of deviance explained) was 38% for present and 34% for future conditions respectively (Table S2, Supplementary Results). Using the 10-fold cross-validation, our top-ranked models (Table S2, Supplementary Results) had a mean prediction error of 19 and 24%, for present and future conditions respectively.

**Figure 3:**
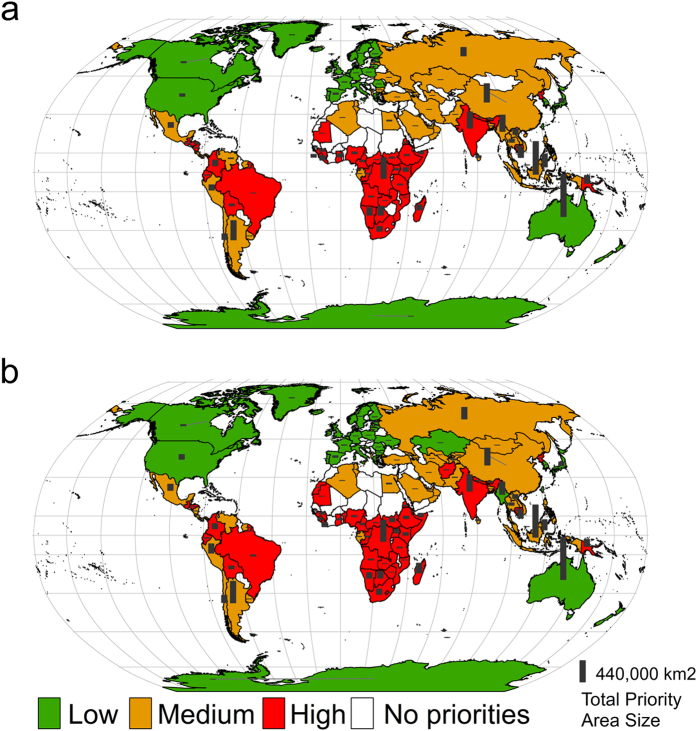
Global risk of human-carnivore conflict. The bars represent the contribution in terms of total area size (km^2^) that each country makes to the 17% protection target for mammalian carnivores under present (a) and future (2040) land use change. No priority means that the country makes no contribution to the 17% protection target. Full details about how the risk index was calculated are available from the Methods section. Figure created in ArcGIS 10.2.1 software (URL http://desktop.arcgis.com/en/).

Presently, South American, African, and South East Asian countries, as well as India, were found to be at highest risk of human-carnivore conflict because of high inequality levels and human development needs (Fig. 3a). In the future, even more countries will be at highest risk of human-carnivore conflict (Fig. 3b).

## Discussion

In this study, we assessed how well carnivores could be represented at the 17% Aichi Target 11 for protection under expected land use change. Our assessment included species-specific requirements for connectivity to create a well-connected conservation landscape for carnivores. We found that land use change will potentially lead to important range losses, particularly amongst already threatened carnivore species. In addition, the land target for protection was found to be inadequate to conserve carnivores under expected land use change. Our results also highlight that land use change will decrease the effectiveness of carnivores to protect other threatened species, especially threatened amphibians. Importantly, countries that will likely make an important contribution to carnivore conservation face important challenges for human and economic development that are likely to further increase direct persecution of carnivores in the future. As such, alternative actions to compliment any proposed expansion of the global protected area network are needed to mitigate human-carnivore conflict outside protected areas. Our results are the most ‘optimistic’ possible, as the 17% land target for protection is here ‘optimally’ allocated for carnivores only. In reality, new protected areas will be identified to represent wider biodiversity, potentially leading to much worse performance, and larger losses of carnivores than we predict.

Our results highlight that the 17% Aichi target for the protection of terrestrial land is inadequate to protect carnivores. This is particularly so for large bodied carnivores, which have the largest habitat requirements and have already experienced substantial population declines and range contractions^13^. Representing all carnivore species more adequately would require a substantial increase in the protection target. However, the indicators of human wealth and development associated with the generalized linear models potentially indicate that the level of resources available for protected area expansion might be lower in countries that were identified as priorities for carnivore conservation. In addition, the funding required to effectively manage already existing protected areas where carnivores are found (e.g. protecting lion requires annual budgets in excess of US$2000/km^2^ 22) might also be inadequate in the same countries. As a result, effectively conserving carnivores in these countries will require a landscape level conservation approach to maintain adequate representation levels for carnivores outside protected areas.

In Europe, the combination of protective legislation, supportive public opinion, and a variety of other practices, is making coexistence between large carnivores and people outside of protected areas possible^23^. Yet, we found that most priorities for carnivore conservation are in developing countries where human populations are increasing in size; agriculture is intensifying; and development needs are the highest. Species that are particularly dangerous to humans, such as lion, highly valued for illegal trade such as tiger, *Panthera tigris*, and sensitive to habitat conversion, such as Ethiopian wolf, *Canis simensis*, and jaguar, *Panthera onca*, are especially vulnerable outside of protected areas^24^,^25^,^26^. While cultural and religious tolerance can facilitate the conservation of carnivores in human-modified landscapes^23^,^27^, the conservation of large predatory carnivores might be more challenging in the absence of sound legislation^28^ and without focussing on the benefits that people derive from carnivores^29^,^30^. First, it will be important to promote and implement strict policies that prevent or mitigate future habitat transformation in priority areas to maintain habitat quality and landscapes permeable to movements of carnivores outside protected areas^16^,^25^,^31^ and across administrative boundaries^28^. Such measures are a priority in countries, such as India, where carnivore species, particularly endemic or near endemic small carnivore species, are especially threatened by habitat transformation. Second, greater efforts to promote tolerance for carnivores should focus on the benefits that people derive from these species^30^. Empirical evidence, in fact, suggests that people’s tolerance for carnivores depends on the perceptions of benefits that carnivores provide^32^,^33^. As a result, promoting the benefits of carnivores as flagships^34^, in controlling the functioning of ecosystems and their resilience to climate change^35^,^36^ and keeping pests under control, could increase tolerance to carnivores in priority areas. Promoting such benefits better will potentially reveal the benefits of protecting rare and elusive small carnivore species that will also be under pressure from agricultural transformation and development in the future.

The benefits provided by carnivore conservation can potentially include the protection of other threatened biodiversity via the umbrella effect of carnivores^37^. Our results potentially show that some of the priority areas for carnivores could be congruent with the location of many threatened species, especially amphibians, which have small and imperiled ranges. At the same time, carnivores were also good surrogates for other mammals, as expected^38^,^39^. However, our results also confirm that protecting carnivores might not deliver efficient conservation solutions for birds and reptiles^40^. While the effectiveness of carnivores as surrogates for terrestrial ecoregions was the lowest among the targeted groups, the priority areas for carnivores might also represent currently less known taxonomic groups that are covered by the ecoregions. Finally, our results suggest that future loss of habitat for carnivores will most likely cause a loss in the representation of many species belonging to other taxonomic groups and terrestrial ecoregions. Enforcing effective conservation actions for carnivores now could therefore prevent future range losses in many other threatened species.

As with previous global conservation planning assessments, a number of caveats need to be highlighted in this study. Species range maps used in this analysis are susceptible to commission errors (when a species is mistakenly thought to be present) and omission errors (when a species is mistakenly thought to be absent)^41^, which may have affected our estimates of carnivore and other biodiversity coverage. However, applying a land use change model to filter the species range maps certainly helped reduce both commission and omission errors because selected areas are less likely to be those where species are absent owing to anthropogenic pressures like habitat loss. Still, anthropogenic factors at the local scale can affect species distributions^42^,^43^. As a result, country-wide or regional conservation planning assessments based on updated species distribution maps that also take into account species-specific responses to human disturbance could be used to refine our results^44^. Future work could also take into account distributional shifts because of climate change. Including a cost layer in the analysis could have been used to identify areas where opportunity costs of conservation are the lowest^45^. At the same time, the land use change model used in this study accounts for regional drivers of change, highlighting areas where pressure on land is the highest. This is particularly important for small-ranged species for which trade-offs to allow development are extremely challenging. In this analysis, we only considered designated protected areas. Including proposed protected areas could show that the representation levels of carnivores within the global protected area network are higher. Finally, more information on how the matrix affects carnivore dispersal could also be included in future regional assessments to identify dispersal corridors.

In conclusion, government, conservation organizations, and donors should act quickly to prevent future loss of carnivore habitats, and to mitigate human-carnivore conflict. A Global Large Carnivore Initiative was started to coordinate local, national, and international research, conservation and policy^13^. Adequate funding is needed to guarantee the enforcement levels required to effectively protect carnivores in already existing protected areas. Meanwhile, our work highlights the need to promote alternative actions outside protected areas in order to enhance carnivore persistence. Promoting interventions based on the socio-economic and political constraints in each priority area might unveil opportunities for carnivore conservation^29^. Losing carnivores from some of the priority areas could eventually lead to cascading effects on species they keep under control^13^ and lead to other biodiversity loss - via the umbrella effect of carnivores, influencing ecosystem functioning, ecosystem services, and human well-being. Our results are available for more detailed examination as interactive maps, graphs and downloadable data to facilitate carnivore conservation and further research (http://avaa.tdata.fi/web/cbig/carnivores).

## Methods

### Carnivore features

We considered a total of 355 species belonging to the orders Carnivora (placental carnivores) and Dasyuromorphia (marsupial carnivores)^5^. The Carnivora is the fifth largest of 29 extant mammalian orders, and includes 282 species in 16 families^5^. Carnivora species occupy almost all terrestrial habitats, as well as many aquatic habitats, from the tropics to the poles^46^. Placental carnivores are distributed across the world, except in Australasia, where the dingo (*Canis lupus dingo*) was most likely introduced by humans^46^. Marsupial carnivores (Dasyuromorphia) are restricted to Australia, Papua New Guinea, Tasmania, and some small nearby islands. They include 73 species in the two extant families of Dasyuridae and Myrmecobiidae^5^.

The analysis included updated species range maps for the Felidae family (http://www.panthera.org/landscape-analysis-lab/maps). The species range data for the other carnivore species were downloaded from the IUCN Red List web site (http://www.iucnredlist.org/)^5^. The IUCN species range maps represent the most frequently updated and publicly available information of the distribution limits of vertebrate species^3^,^47^. However, they may overestimate the species’ true area of occupancy, because, for example, they include areas from which the species is absent, such as large freshwater bodies for terrestrial species^48^. As a result, we refined the species range maps by accounting for present and future land use allocation, as we describe below.

### Global land use change scenarios

We used global land use change scenarios developed independently of this study^21^. In the models, land use changes are driven by macro-economic assessment of regional demand and supply of agricultural commodities^49^, accounting for local factors that either promote or constraint land use change^21^. The scenario is based on the OECD Environmental Outlook to 2050^50^. Land availability, as well as socio-economic and biophysical conditions, steer the model to convert land use systems, either resulting in a predicted expansion of human dominated land use systems over semi-natural systems, or leading to a predicted intensification of land management to fulfill world-region scale demands. As a result, the model provides a good representation of the multiple drivers of habitat loss. At the same time, the model simulates abandonment of agricultural practices and re-wilding, which were found to be important factors in the recovery of large carnivores in Europe^23^.

The land use change models for 2000, representing the present, and 2040, representing the future, were reclassified into a condition layer to account for habitat quality and degradation of each land use system^11^. The condition values vary between 0 and 1.0 where a value of 0 indicates a completely degraded condition and a value of 1.0 indicates pristine condition. All natural land use systems in the land use change model (e.g. forests and natural grasslands) have a value of 1.0. While these values were a reasonable first approximation based on published literature^11^, the same effects across all species were assumed, as information on the effects of different land uses on each species is currently not available^51^. To reduce uncertainty on the impact that land use change will have on the different species, we accounted for both an optimistic and a pessimistic scenario. Scenario planning offered a framework for exploring the uncertainty surrounding the future consequences of land allocation and potential responses of different carnivore species to different land uses. Under the pessimistic scenario, all intensive land use systems (e.g. cropland intensive)^21^ were assumed to be detrimental to carnivores, and assigned a value of 0^11^. The values in the original species range maps were then multiplied by the condition values for present and future (optimistic and pessimistic) scenarios, respectively, in the spatial conservation prioritization software Zonation^19^,^20^. In the Zonation analyses, we used the transformed sets of species range maps for the present and future (optimistic and pessimistic) scenarios, respectively.

### Spatial conservation prioritization

In order to identify the priority areas for carnivore conservation, we used the Zonation version 4.0 software^52^,^53^. Compared to other conservation planning tools, Zonation produces a complementarity-based and balanced ranking of conservation priority over the entire landscape^19^,^20^, rather than satisfying specific targets at minimum cost. The priority ranking is produced by iteratively removing the grid cell or planning unit that leads to smallest aggregate loss of conservation value, while accounting for total and remaining distributions of features, weights given to species, and species-specific connectivity. How loss of conservation value occurring in a cell is aggregated across features depends on the so-called ‘cell-removal rule’. Detailed explanations about Zonation are provided in^52^,^53^.

All input data were rasterized to global high resolution grids (0.0083 degrees) in a latitude/longitude coordinate system. The analysis extent was masked to terrestrial land only. The additive-benefit function formulation for aggregation of conservation value was used^19^. Species were weighted proportionally to their IUCN Red List category (http://www.iucnredlist.org/technical-documents/categories-and-criteria). Specifically, we assigned a weight = 1 to species of Least Concern; 2 to Near Threatened species; 3 to Vulnerable and Data Deficient species; 4 to Endangered species; and 5 to Critically Endangered species. Data Deficient species were assigned the same weight as vulnerable species, in line with the precautionary principle^54^.

Finally, we accounted for connectivity in the form of species-specific home-range requirements, which may be critical for carnivores^16^. This was done so by using a species-specific connectivity method (distribution smoothing) that emphasizes areas of high habitat quality and density^52^. Particularly, this connectivity method favors uniform areas as opposed to fragmented ones. The connectivity of cells is determined with a smoothing kernel, where the radius of the kernel was approximated as the radius of the home-range for each species. Consequently, cells that are surrounded by many occupied cells within the home-range radius receive a higher value than isolated cells. The home-range sizes for each species were obtained from^46^. Full details on how to account for species-specific connectivity in Zonation are provided in^52^.

Zonation automatically produces a number of different output files for each run^52^. In this study, we discuss only the most relevant outputs. In the priority rank map, each grid cell has a value between 0 and 1, meaning that values close to 0 were removed first because of their low conservation value and priority, while high values close to 1 were retained until the end to reflect their high conservation value and priority. The priorities are derived from the order of iterative cell ranking, or removal. Performance curves quantify the proportion of the range maps retained for each species, at each top fraction of the landscape chosen for conservation. The performance curves correspond directly with the priority rank map. Zonation outputs can also be visualized, for example, by using parallel boxplots to display the median, quartiles, and minimum and maximum of original total range remaining across a set of species or groups, calculated for a specific priority top fraction of the landscape (e.g., 17% of the priority rank map). Finally, all results were visualized for two scenarios only (present and future). For the future scenarios, we averaged the outputs of the optimistic and pessimistic scenarios into one future consensus scenario.

Under each analysis, we carried out a gap analysis to consider how well the global protected area network represents carnivores, and we also identified the priority areas for expanding the protected area network. The data on protected areas was extracted from the World Database on Protected Areas (http://www.protectedplanet.net). We selected only protected areas belonging to IUCN protected area categories I to VI, and having as status ‘designated’. These areas covered approximately 11% of the Earth’s land surface (including Antarctica)^11^.

### Surrogacy analyses

We assessed the effectiveness of 341 carnivore species as surrogates for 23,110 other vertebrate species and 867 terrestrial ecoregions. We based our surrogacy analysis on fully assessed species (i.e., we left out Data Deficient species) included in the IUCN Red List (IUCN 2014). We downloaded the species range data for mammals, amphibians and reptiles from the Spatial Data Download area of the IUCN Red List web site (http://www.iucnredlist.org/). Data for birds were obtained from the BirdLife International’s Data Zone page (http://www.birdlife.org/datazone/home). Data for terrestrial ecoregions, which are large units of land containing a geographically distinct assemblage of species, natural communities, and environmental conditions, were downloaded from the World Wildlife Fund conservation science data page (http://www.worldwildlife.org/publications/terrestrial-ecoregions-of-the-world). In order to assess the effectiveness of carnivores as surrogates for other vertebrate classes, we assigned a weight of 0.0 to all other vertebrate species and terrestrial ecoregions in Zonation. Hence, only the carnivore species were influencing the prioritization, while it was possible to track their performance in representing the targeted species. Full details about surrogacy analyses in Zonation can be read in^40^.

### Risk index

In ArcGIS (v. 10.1), we calculated the proportion of each country falling within the 17% land target, by dividing the total area size of each country falling within the 17% land target by the total area size of the 17% land target under both the present and future scenarios. We then ran generalized linear models with a negative binomial error distribution, accounting for overdispersion, and a log-link function, to examine the socio-economic and political factors potentially affecting carnivore conservation within each country. The response variable was the proportion of each country falling within the 17% land target. As predictor variables, we used socio-economic variables that are normally used in such analyses (Table S3 in Supplementary Methods)^55^,^56^. Following Spearman’s rank correlations, we only retained the predictor variables with the greatest explanatory effect that were not strongly correlated.

We used an information theoretic approach^57^ and Akaike’s information criterion weights to assess each model’s relative probability, and its structural goodness of fit using the percentage of deviance explained by the model. We determined the magnitude and direction of the coefficients for the independent variables with multi-model averaging implemented in the R (version 3.1.0)^58^ package glmulti^59^. The relative importance of each predictor variable was measured as the sum of the Akaike weights over the 6 top-ranked models containing the parameter of interest^60^. Finally, we validated the top-ranked model by using leave-one out cross validation, which is used to estimate the mean model-predictor error by successively omitting 1 observation from the training data set and using it for validation.

After running the generalized linear models, we developed a country risk index by using the arithmetic mean of the most important predictor variables (relative importance ≥0.7), and the proportion of each country falling within the 17% global land target. We then classified each country into low, medium and high risk by using tertiles (low risk for the lower part; medium risk for the medium part; and high risk for the higher part). A tertile is any of the two points that divide an ordered distribution into three parts, each containing a third of the population.

## Additional Information

**How to cite this article**: Di Minin, E. *et al.* Global priorities for national carnivore conservation under land use change. *Sci. Rep.* **6**, 23814; doi: 10.1038/srep23814 (2016).

## Supplementary Material

Supplementary Information

## Figures and Tables

**Table 1:** Large-bodied carnivores, shown in open cells, and small-bodied carnivores shown in shaded cells, that will suffer the most extensive range losses under opposing scenarios of present and future land use change.

Common name	Scientific name	Family	Status & pop. trend	Present – Prop Rem	Future – Ranking & Prop Rem	Geographic range
1) Sloth bear	*Melursus ursinus*	Ursidae	VU ↓	0.575	↔ 1 (0.335)	India; Nepal; Sri Lanka; Bhutan
2) Red Wolf	*Canis rufus*	Canidae	CE ↑	0.624	↔ 2 (0.606)	USA
3) Sunda Clouded Leopard	*Neofelis diardi*	Felidae	VU ↓	0.742	↓ 9 (0.727)	South East Asia
4) Ethiopian wolf	*Canis simensis*	Canidae	EN ↓	0.783	↑ 3 (0.650)	Ethiopia
5) Dhole	*Cuon alpinus*	Canidae	EN ↓	0.805	↑ 4 (0.666)	Central and Eastern Asia
6) Asiatic Black Bear	*Ursus thibetanus*	Ursidae	VU ↓	0.859	↓ 8 (0.723)	Asia
7) Striped Hyaena	*Hyaena hyaena*	Hyaenidae	NT ↓	0.861	↓ 13 (0.792)	Africa & Asia
8) Clouded Leopard	*Neofelis nebulosa*	Felidae	VU ↓	0.873	↑ 7 (0.722)	South East Asia
9) Gray wolf	*Canis lupus*	Canidae	LC ↔	0.877	↓ 14 (0.813)	North America, Europe, Asia
10) African Clawless Otter	*Aonyx capensis*	Mustaelidae	LC ↔	0.881	↑ 6 (0.721)	sub-Saharan Africa
11) Tiger	*Panthera tigris*	Felidae	EN ↓	0.887	↓ 12 (0.773)	Asia
12) Giant Panda	*Ailuropoda melanoleuca*	Ursidae	EN ↓	0.893	↑ 5 (0.716)	China
15) Leopard	*Panthera pardus*	Felidae	NT ↓	0.894	↑ 11 (0.771)	Africa & Asia
16) Spotted Hyena	*Crocuta crocuta*	Hyaenidae	LC ↓	0.897	↑ 10 (0.766)	sub-Saharan Africa
1) Javan Ferret Badger	*Melogale orientalis*	Mustelidae	DD ?	0.417	↓ 11 (0.364)	Indonesia
2) Malabar Civet	*Viverra civettina*	Viverridae	CE ?	0.453	↔ 2 (0.258)	India
3) Ruddy Mongoose	*Herpestes smithii*	Herpestidae	LC ↓	0.469	↓ 5 (0.268)	India, Sri Lanka
4) Rusty-spotted Cat	*Prionailurus rubiginosus*	Felidae	VU ↓	0.476	↑ 3 (0.259)	India, Sri Lanka
5) Stripe-necked Mongoose	*Herpestes vitticollis*	Herpestidae	LC ↔	0.481	↑ 4 (0.265)	India, Sri Lanka
6) Bengal Fox	*Vulpes bengalensis*	Canidae	LC ↓	0.495	↔ 6 (0.314)	Bangladesh; India; Nepal; Pakistan
7) Egyptian Weasel	*Mustela subpalmata*	Mustelidae	LC ↔	0.518	↓ 17 (0.452)	Egypt
8) Indian Grey Mongoose	*Herpestes edwardsii*	Herpestidae	LC ?	0.522	↑ 7 (0.345)	Asia
9) Indian Brown Mongoose	*Herpestes fuscus*	Herpestidae	VU ↓	0.532	↑ 8 (0.346)	India, Sri Lanka
10) Brown Palm Civet	*Paradoxurus jerdoni*	Viverridae	LC ?	0.541	↔ 10 (0.363)	India
11) Nilgiri Marten	*Martes gwatkinsii*	Mustelidae	VU ↓	0.546	↓ 25 (0.499)	India
13) Red-tailed Phascogale	*Phascogale calura*	Dasyuridae	NT ↓	0.567	↑ 9 (0.351)	Australia
48) Harris’s Olingo	*Bassaricyon lasius*	Procyonidae	DD ↓	0.768	↑ 12 (0.390)	Costa Rica
77) Subtropical Antechinus	*Antechinus subtropicus*	Dasyuridae	LC ↔	0.818	↑ 1 (0.237)	Australia

Non-consecutive numbers in the first column correspond to species that are presently not in the top 11 species that suffered the largest range loss, but will be in the top 11 under future land use change. Prop Rem is the proportion of original range remaining. Ranking starts from the species losing more range. Arrows indicate change in rank. Additional information on species’ conservation status, population trend and range was retrieved from: http://www.iucnredlist.org/.
