# Supplementary material for: Global priorities for national carnivore conservation under land use change
Source: Sci Rep. 2016 Apr 1;6:23814. doi: 10.1038/srep23814 (PMC4817124; doi:10.1038/srep23814)
Supplement: Supplementary Information [file srep23814-s1.pdf]

Global priorities for national carnivore conservation under land use change

Enrico Di Minin [1,2*], Rob Slotow [2, 3], Luke T.B. Hunter [2,4], Federico Montesino Pouzols [1, 5], Tuuli Toivonen [1, 6], Peter H. Verburg [7], Nigel Leader-Williams [8], Lisanne Petracca [4], Atte Moilanen [1]

1 Department of Biosciences, University of Helsinki, FI-00014, Helsinki, Finland; 2 School of Life Sciences, University of KwaZulu-Natal, Durban 4041, South Africa; 3 Department of Genetics, Evolution and Environment, University College, London, United Kingdom; 4 Panthera, New York, New York, United States of America; 5 Rutherford Appleton Laboratory, Science & Technology Facilities Council, Harwell Oxford Campus, Didcot OX11 0QX, UK; 6 Department of Geography, University of Helsinki, FI-00014, Helsinki, Finland; 7 Faculty Earth and Life Sciences, VU University Amsterdam, De Boelelaan 1087, Amsterdam, 1081 HV, The Netherlands; 8 Department of Geography, University of Cambridge, Downing Place, Cambridge, CB2 3EN, UK.

*Corresponding author: Email: enrico.di.minin@helsinki.fi

Supplementary Appendix S1

Results

Table S1 Proportion of range lost due to land-use change from present to future (2040) conditions.

Values are reported for carnivore species grouped by IUCN status, order and body size.

Group of species	Present	Future
All	0.084	0.179
Least Concern	0.075	0.182
Near Threatened	0.085	0.159
Vulnerable	0.082	0.222
Endangered	0.066	0.113
Critically Endangered	0.150	0.186
Data Deficient	0.074	0.180
Carnivora	0.090	0.195
Dasyuromorphia	0.051	0.079
Small-bodied	0.086	0.159
Large-bodied	0.077	0.178

Table S2 Top-ranked predictors of the contribution of each country to carnivore conservation.

Present								
Model		AIC	Delta	Weight	% of deviance explained	Cross-validation	Variable	Relative importance
1	extpres ~ hdi + gini	276.80	0.00	0.33	0.38	0.19	gini	1.00
2	extpres ~ hdi + gini + popdens	278.03	1.23	0.18	0.38		hdi	0.82
3	extpres ~ hdi + gini + prot	278.55	1.76	0.14	0.34		cpi	0.23
4	extpres ~ gini + cpi	278.75	1.95	0.13	0.38		popdens	0.18
5	extpres ~ hdi + gini + cpi	278.88	2.09	0.12	0.33		prot	0.12
6	extpres ~ gini	279.04	2.24	0.11	0.35			
Future								
Model		AIC	Delta	Weight	% of deviance explained	Cross-validation	Variable	Relative importance
1	extfut ~ hdi + gini	300.60	0.00	0.32	0.34	0.23	gini	1.00
2	extfut ~ gini + cpi	301.42	0.82	0.21	0.35		hdi	0.70
3	extfut ~ hdi + gini + cpi	302.32	1.72	0.13	0.33		cpi	0.35
4	Extfut ~ hdi + gini + popdens	302.58	1.98	0.12	0.35		popdens	0.11
5	extfut ~ hdi + gini + prot	302.74	2.14	0.11	0.34		prot	0.11
6	extfut ~ gini	302.80	2.20	0.11	0.36			

Notes: the tildes (~) imply a correlative relationship between the predictors and the response variable (proportion of the country within the top 17% target for carnivore conservation). Abbreviations: AIC, Akaike's information criterion; hdi = human development index; cpi = corruption perception index; popdens = human population density; gini = Gini index; prot = proportion of the country protected.

Figure S1. Box-and-whisker plots showing the representation levels for all carnivore species at the 17% target for the expansion of the protected area network, as listed by the International Union for the Conservation of Nature. LC = least concern; TH = near threatened; VU = vulnerable; EN = endangered; CE = critically endangered; DD = data deficient.

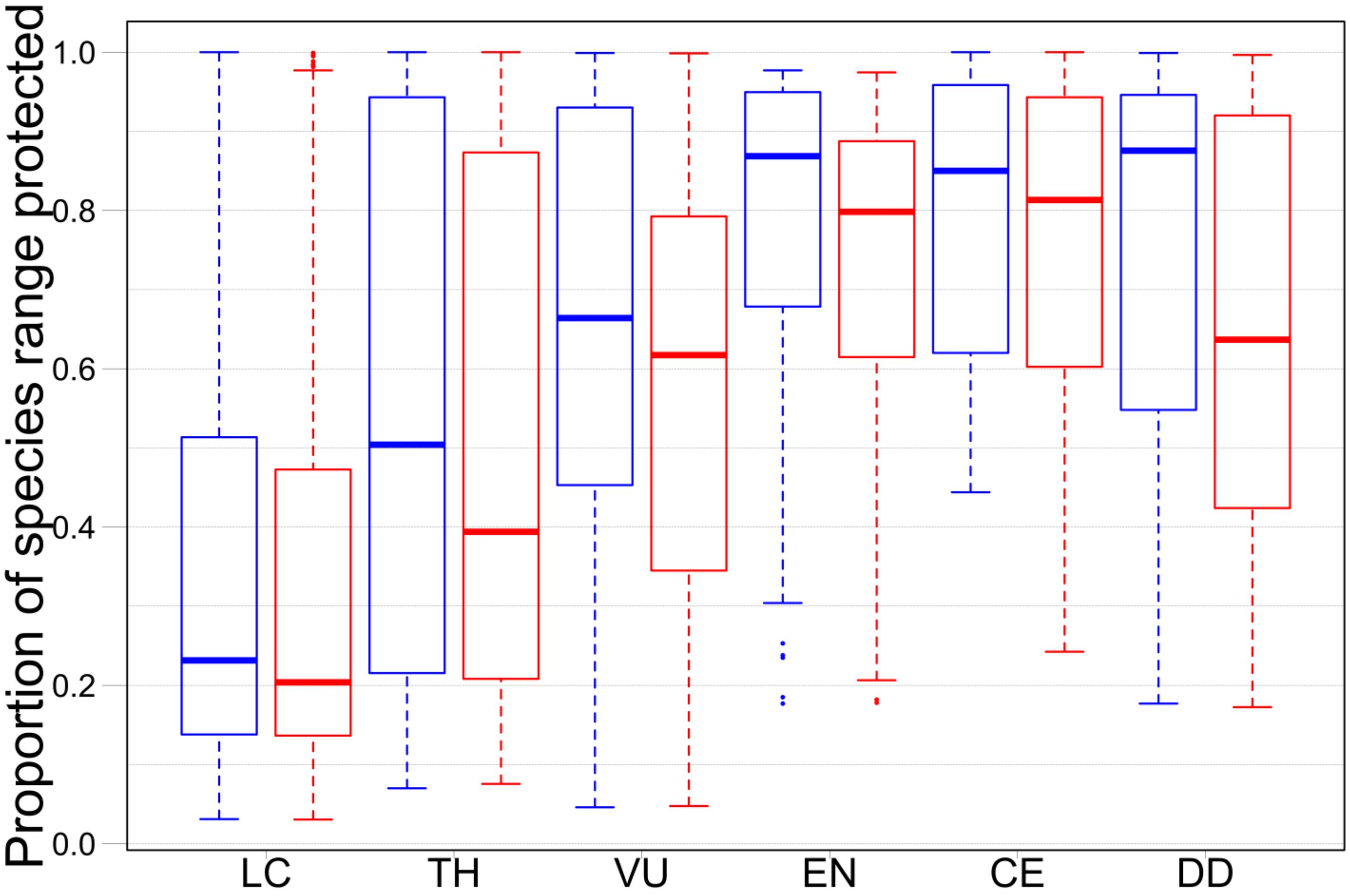

Figure S2. Box-and-whisker plots showing the representation levels for each family belonging to placental and marsupial carnivores at the 17% target for the expansion of the protected area network.

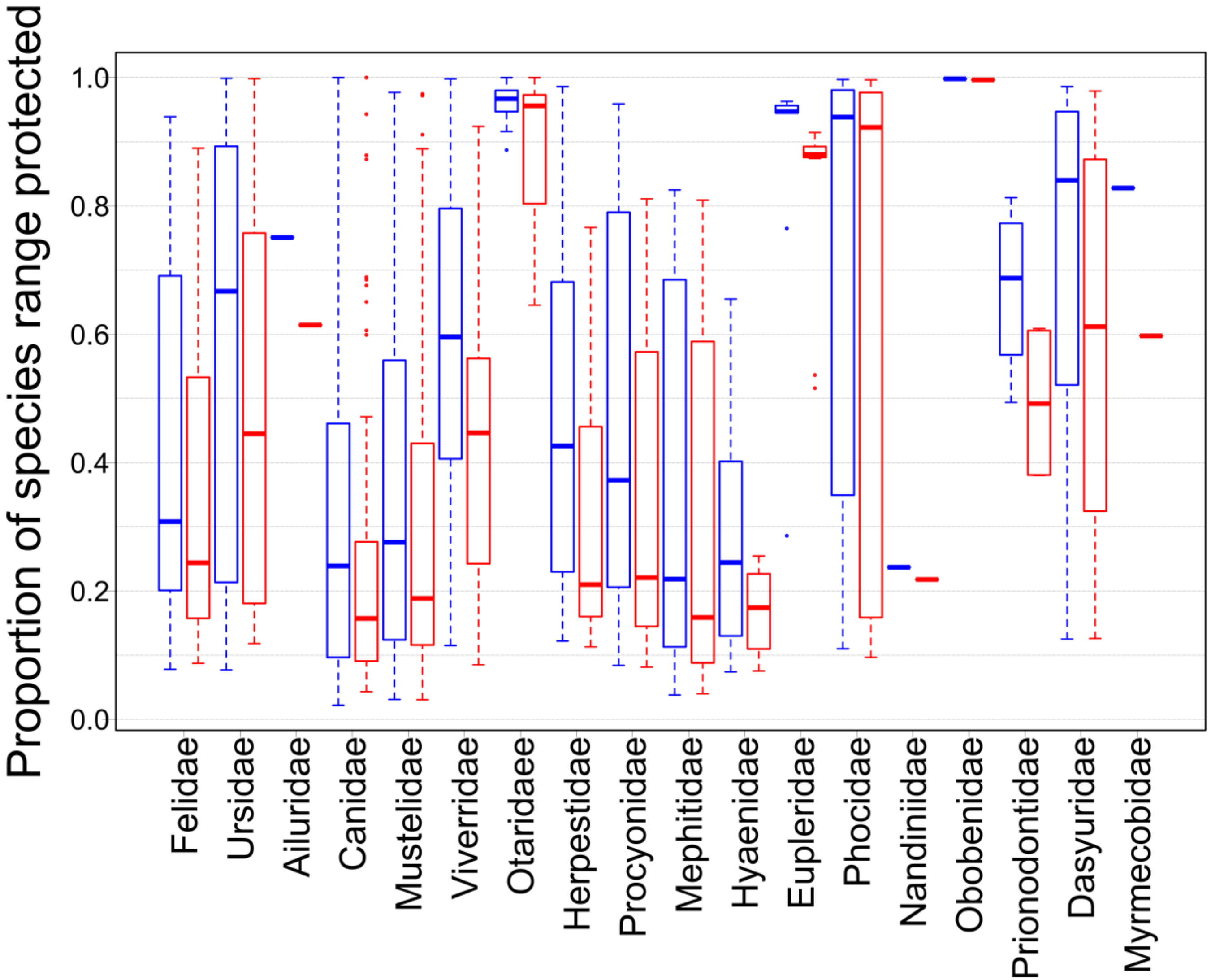

Figure S3. Box-and-whisker plots showing the representation levels for large and small-bodied carnivores at the 17% target for the expansion of the protected area network.

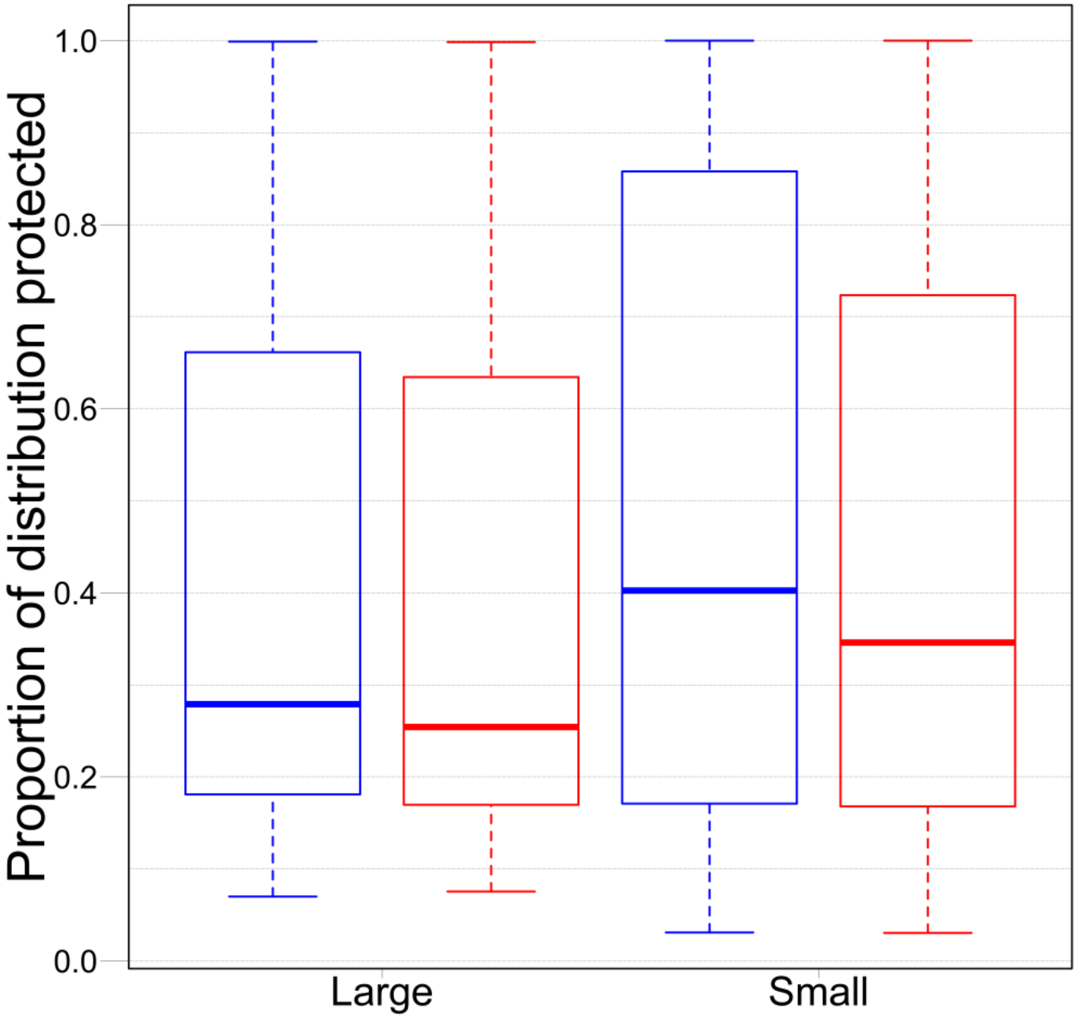

Figure S4. Box-and-whisker plots showing the representation levels for placental (Carnivora) and marsupial (Dasyuromorphia) carnivores at the 17% target for the expansion of the protected area network.

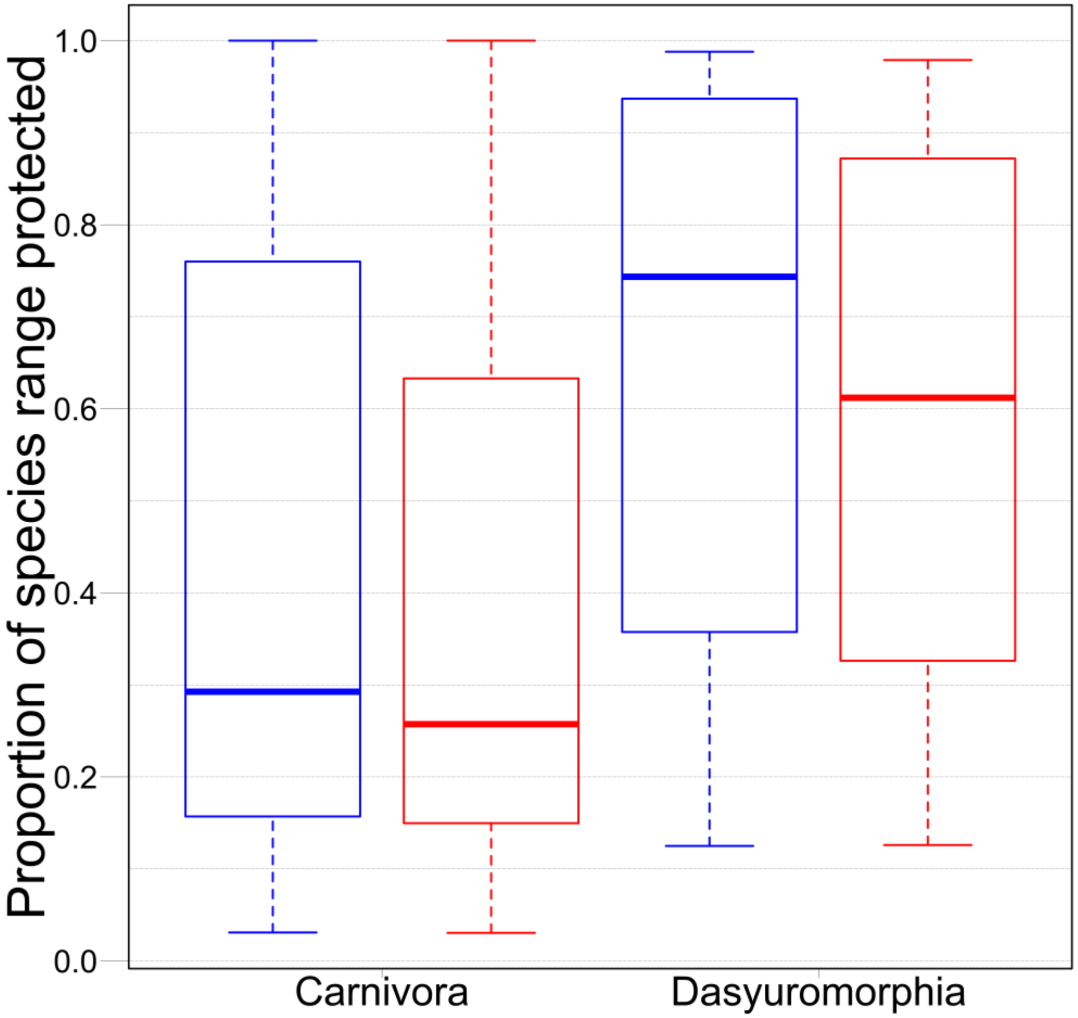

Figure S5. Box-and-whisker plots showing carnivore surrogacy effectiveness at 17% of all terrestrial land protected for (a) amphibians, reptiles, birds, mammals, and ecoregions, and (b) amphibians, reptiles, birds, mammals as listed by the International Union for the Conservation of Nature (LC = least concern; NT = near threatened; VU = vulnerable; EN = endangered; CE = critically endangered), under present (blue), and future (2040, red), land use allocation.

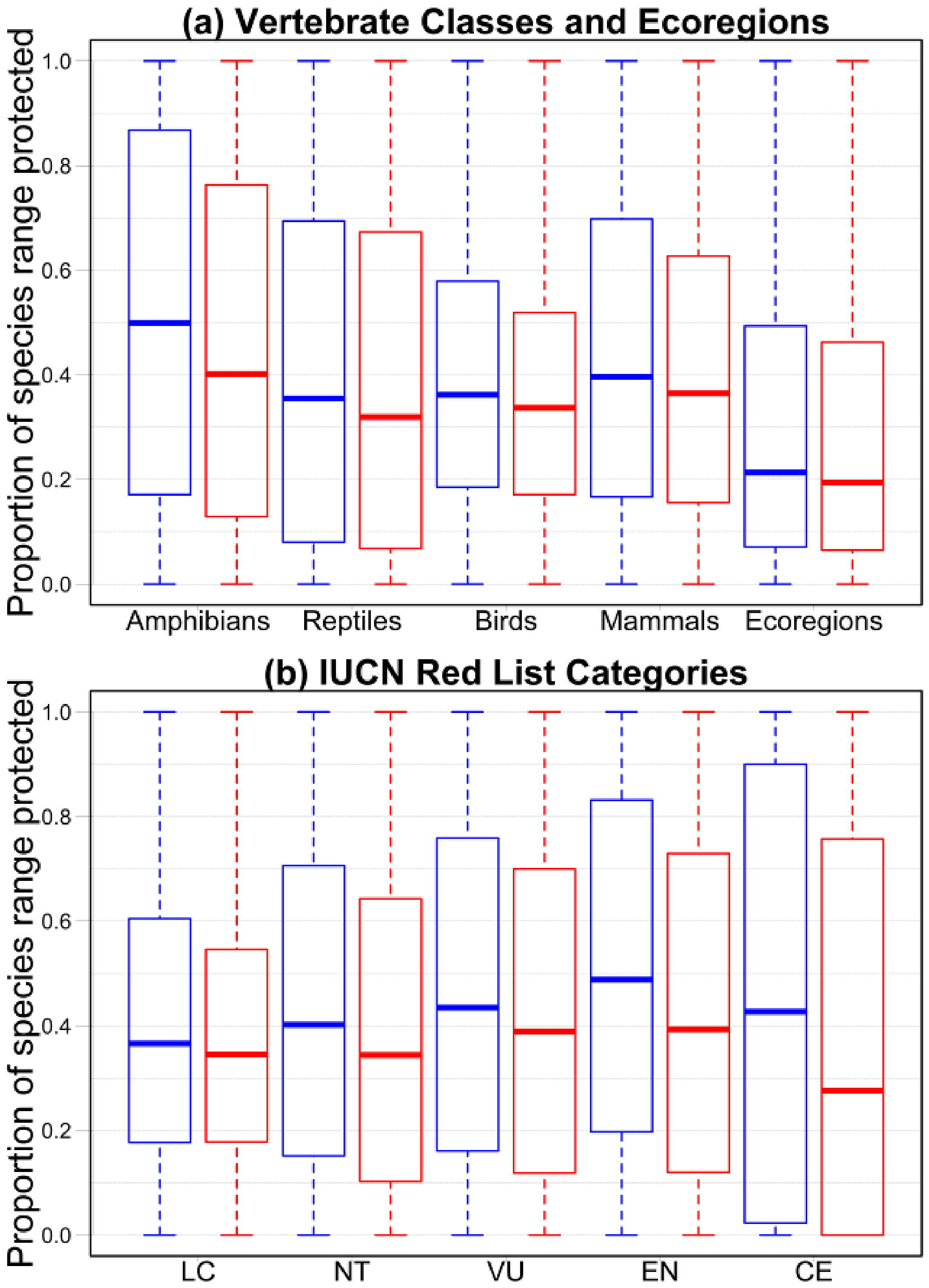

Figure S6. Model-averaged coefficients and standard errors showing the relative effects of five variables on the proportion of a country within the top 17% target for carnivore conservation. Numbers on the bars are the p-values for the significant variables. (a) Present proportion of a country within the top 17% target for carnivore conservation was used as the response variable. (b) Future proportion of a country (based on a consensus scenario) within the top 17% target for carnivore conservation was used as the response variable. Abbreviations: hdi = human development index; cpi = corruption perception index; popdens = human population density; gini = Gini index; prot = proportion of the country protected. For details and interpretation of the variables, see Table S3 below. The dispersion parameter in (a) was 1.861 and in (b) 1.792.

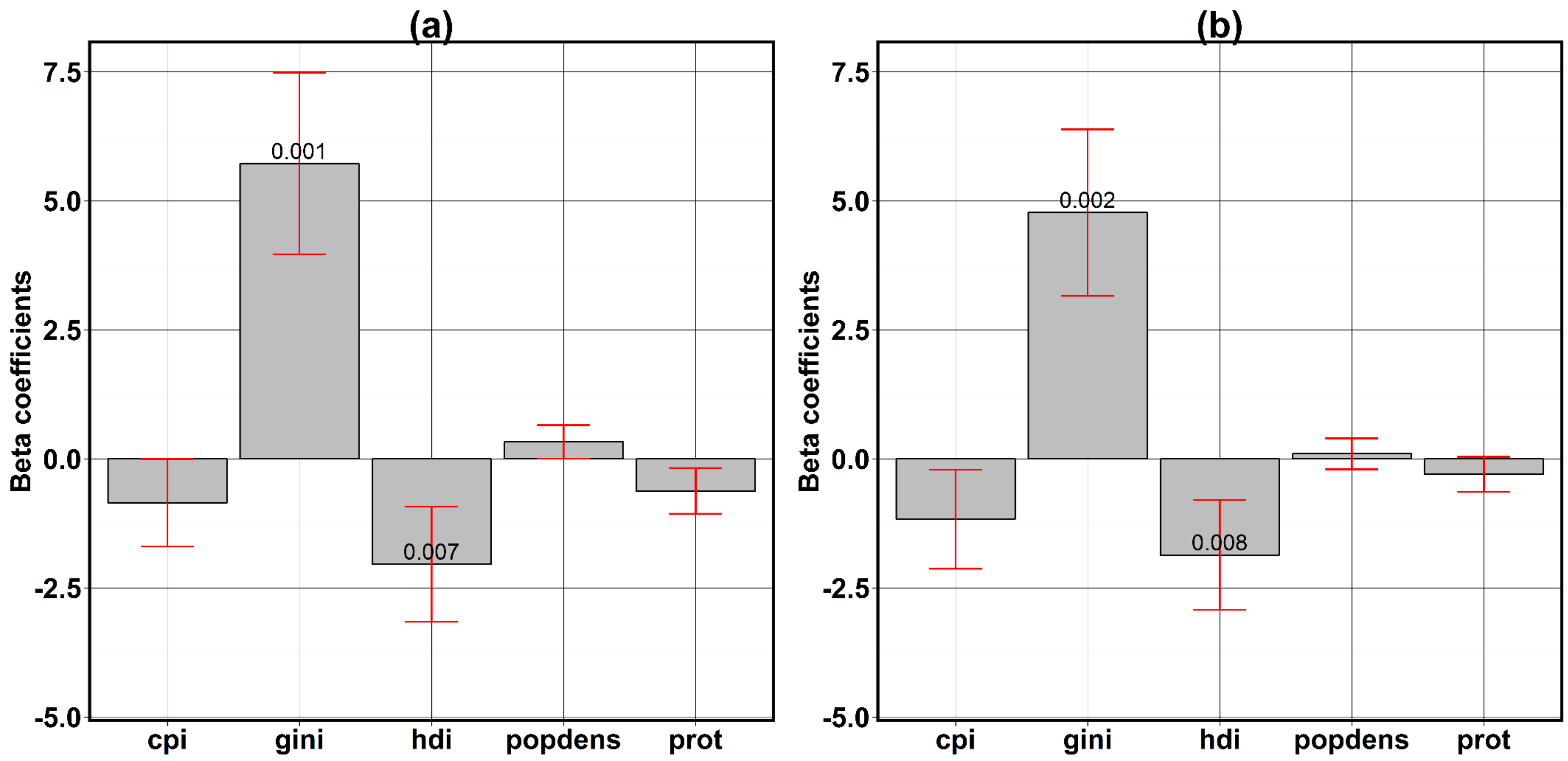

Methods

Table S3. List of predictor variables used in the generalized linear models, their description, unit, year of the data and source of the data.

Variable	Description	Unit	Year	Source
The Human Development Index	Composite index of human development: a long and healthy life; being knowledgeable; and have a decent standard of living	From 0 = least developed, to 1 = most developed	2014	http://hdr.undp.org/en/content/human-development-index-hdi
GINI index	The extent to which the distribution of income/consumption expenditure among individuals/households within an economy deviates from a perfectly equal distribution	From 0 = perfect equality, to 100 = perfect inequality	2013	http://data.worldbank.org/indicator/SI.POV.GINI?page=1
Corruption perception index	An index measuring the perceived level of corruption in the public sector of a country	From 0 = highly corrupt, to 100 = least corrupt	2014	http://www.transparency.org/research/cpi/overview
Population density	The density of the population in each country - Present	No. People per km^2	2014	http://data.worldbank.org/indicator/EN.POP.DNST
Population density	The density of the population in each country - Future	No. People per km^2	2050	http://www.unfpa.org/pds/trends.htm
Protection	Proportion of terrestrial land currently protected	% of total land area	2012	http://data.worldbank.org/indicator/ER.LND.PTLD.ZS
